# MEDIATING ROLE OF DEPRESSION IN THE ASSOCIATION BETWEEN CUMULATIVE LONELINESS AND MEMORY FUNCTION, 2006–2020

**DOI:** 10.1093/geroni/igae098.0285

**Published:** 2024-12-31

**Authors:** Xuexin Yu, Laura Zahodne, Alden Gross, Belinda Needham, Kenneth Langa, Lindsay Kobayashi

**Affiliations:** University of Michigan, Ann Arbor, Michigan, United States; University of Michigan, Ann Arbor, Michigan, United States; Johns Hopkins Bloomberg School of Public Health, Baltimore, Maryland, United States; University of Michigan, Ann Arbor, Michigan, United States; University of Michigan, Ann Arbor, Michigan, United States; University of Michigan, Ann Arbor, Michigan, United States

## Abstract

**Objective:**

To investigate the mediating effect of depressive symptoms in the association between cumulative loneliness and memory function.

**Methods:**

Data were from 4,764 adults aged 50+ in the Health and Retirement Study in two random sub-cohorts from 2006-2018 (Cohort A) and 2008-2020 (Cohort B). Participants were categorized as experiencing loneliness at 0, 1, 2, or 3 time points according to the UCLA Loneliness Scale, administered every four years from 2006-2014 (Cohort A) or 2008-2016 (Cohort B), creating an 8-year exposure period in each cohort. Depressive symptoms were measured using the Center for Epidemiologic Studies Depression Scale in 2016 (Cohort A) or 2018 (Cohort B). Episodic memory function was assessed in 2018 (Cohort A) or 2020 (Cohort B). Causal mediation analysis was performed to investigate the mediating role of depressive symptoms in the cumulative loneliness-memory association, in the pooled cohorts.

**Results:**

Mean age (SD) at baseline was 65 (7.6), and 62% were female (2,934/4,764). Greater cumulative loneliness over the 8-year exposure period was associated with lower memory function at the follow-up in a dose-response relationship. This association was mediated by depressive symptoms, with natural indirect effects ranging from -0.009 (95% CI: -0.016 to -0.002, loneliness at one time point), -0.016 (95% CI: -0.028 to -0.003, loneliness at two time points), to -0.020 (95% CI: -0.037 to -0.004, loneliness at three time points, 20% mediated).

**Discussion:**

Depression could be a psychological mechanism through which cumulative loneliness affects memory function among middle-aged and older adults in the United States.

